# Oil Stone for Sharpening Drills

**Published:** 1904-11

**Authors:** 


OIL STONE FOR SHARPENING DRILLS.—With the use of a piece of oilstone shaped like a pocket-knife blade (say three inches in length and with a sharp edge), it is a comparatively easy matter to bring in turn each blade of the drill to an edge, examining the drill frequently during the process under a magnifying glass. It is essential to good work that the stone edge be kept sharp. To accomplish this, obtain a piece of hardwood say three inches long and two inches in width, making sure that it is straight and perfectly level. Now take a strip of sheet lead two inches wider than the wood, but the same length or longer, so that it may be continued over each end; tack it over the sides and ends (if necessary) to the wood, after having adjusted it evenly. Upon the surface thus formed sprinkle some No. 1 emery, and rub the stone lengthwise, without oiling or wetting the surface. To keep the stone in perfect condition this process should be continued after every dozen drills. As to the time required for sharpening the stone, three minutes usually suffices.

When utterly worn out the drills can still be made to render useful service by grinding them to a spear-point upon the lathe stone. In this shape they are very useful in alveolar abscess, where it is to be approached from the outside, through the alveolar process.—*Cosmos.*

